# Deciphering Seedling-Stage Salinity Stress Tolerance in Maize Genotypes Through Morpho-Physiological and Ionic Traits

**DOI:** 10.3390/ijms27073037

**Published:** 2026-03-26

**Authors:** Pardeep Kumar, Vineeth T. V., Shyam Bir Singh, Mukesh Choudhary, Bhupender Kumar, Anuj Kumar, Sujay Rakshit, Hanuman Sahay Jat

**Affiliations:** 1 ICAR-Indian Institute of Maize Research, Ludhiana 141008, India; pardeepkumar656@gmail.com (P.K.); singhsb1971@gmail.com (S.B.S.); anuj2011.ag@gmail.com (A.K.); hsjat_agron@yahoo.com (H.S.J.); 2 ICAR-Central Soil Salinity Research Institute, Bharuch 392012, India; vinee2705@gmail.com; 3 The Institute of Agriculture, The University of Western Australia, Crawley, WA 6009, Australia; 4 ICAR-Indian Institute of Agricultural Biotechnology, Ranchi 834003, India; s.rakshit@icar.gov.in

**Keywords:** maize, morpho-physiological traits, salinity, stress indices, K^+^/Na^+^ ratio

## Abstract

Salinity stress impairs maize growth by inducing osmotic stress, pigment degradation, and ionic imbalance, particularly during early seedling development. This study investigated the morpho-physiological and ionic responses of different maize genotypes exposed to increasing salinity levels (control, 3, 6, and 9 dS/m) at the seedling stage. Salinity caused a reduction in biomass accumulation (shoot fresh weight and shoot dry weight), plant height, and K^+^/Na^+^ ratio, with pronounced effects under severe stress. Significant genotypic variability was detected for photosynthetic pigments (chlorophyll a, chlorophyll b, total chlorophyll and carotenoids) growth traits, and ionic regulation, indicating diverse physiological adaptation strategies. Stress tolerance indices and multivariate analysis revealed that chlorophyll stability, carotenoid accumulation, and maintenance of ionic homeostasis (K^+^/Na^+^ ratio) were the dominant physiological determinants of salinity tolerance. Additionally, principal component analysis showed a shift from biomass-driven variation under non-stress conditions to pigment- and ion-driven variation under higher salinity. Based on the results, genotypes BML 6 and HKI 163 maintained higher pigment content and improved K^+^/Na^+^ balance, enabling better growth under saline conditions. These findings highlight key physiological traits underlying salinity tolerance and provide insight into early-stage adaptive mechanisms in maize.

## 1. Introduction

Maize (*Zea mays* L.) is one of the most important cereal crops worldwide after wheat and rice and plays a pivotal role in global food, feed, and industrial systems [1]. Domesticated from its wild ancestor teosinte in Mexico approximately 7000 years ago [2], maize has evolved into a highly adaptable crop cultivated across diverse agro-climatic regions. It serves as a raw material for numerous industrial products and constitutes a major component of livestock feed [3,4,5]. Currently, maize is grown across approximately 208 million hectares globally, producing nearly 1241 million metric tons, with an average productivity of about 5.96 tons per hectare (FAOSTAT, 2023). In South Asia, maize cultivation is expanding rapidly, particularly within rice-based cropping systems, owing to its high yield potential and favorable economic returns [5,6]. Of the total global maize production, approximately 61% is utilized as animal feed, 17% for direct human consumption, and 22% for industrial applications, underscoring its multifaceted importance in global agricultural systems [7]. Ensuring sustainable maize production is therefore critical for food security, livestock sustainability, and rural livelihoods [8]. In India, maize production reached 44.3 million tons from 12.09 million hectares during 2024–25, reflecting notable improvements in productivity (https://upag.gov.in/). India contributes nearly 3% of global maize production and ranks fifth worldwide after the United States, China, Brazil, and Argentina (https://www.fas.usda.gov/). With the global population projected to reach approximately 9.5 billion by 2050 [5,9,10], the demand for maize is expected to increase substantially. However, expanding production is increasingly constrained by land degradation, soil fertility decline, and climate variability [11]. Meeting future demand will therefore require not only area expansion but also significant improvements in productivity under suboptimal environmental conditions.

Among abiotic stresses, soil salinity is one of the most serious constraints affecting agricultural productivity, particularly in arid and semi-arid regions where irrigation-induced salinization and poor-quality groundwater are prevalent. Globally, more than 800 million hectares of land are affected by salinity, posing a major threat to sustainable crop production [12,13]. Approximately 45 million hectares of irrigated land have already been degraded due to salt accumulation, and an additional 1.5 million hectares are lost annually as a result of progressive salinization [14]. Currently, nearly 20% of the world’s cultivated land is affected by salinity, and projections suggest that this proportion may increase dramatically in the coming decades, potentially impacting up to 50% of agricultural land by 2050 [15,16]. These trends represent a serious challenge to global food security and agricultural sustainability.

Soils with an electrical conductivity (EC) of 4 dS/m or higher, or an exchangeable sodium percentage (ESP) of 15%, are generally classified as saline [17]. However, yield reductions may occur even at lower EC levels depending on crop sensitivity [4]. Maize is considered moderately sensitive to salinity, with a threshold soil electrical conductivity (ECe) of 1.7 dS/m. Beyond this threshold, grain yield declines by approximately 12% for each unit increase in ECe [4]. Salt stress affects maize through two primary mechanisms: osmotic stress and ion toxicity. Osmotic stress occurs due to reduced soil water potential around the root zone, limiting water uptake and impairing cell expansion [18]. Subsequently, excessive accumulation of sodium (Na^+^) in plant tissues leads to ion toxicity, metabolic disruption, premature senescence, and reduced growth [19]. These processes interfere with nutrient balance, cellular integrity, and key physiological functions such as photosynthesis and respiration [20].

Plant adaptation to salinity involves complex physiological and biochemical mechanisms aimed at maintaining ionic and osmotic balance. A critical component of salt tolerance is the maintenance of ionic homeostasis through selective ion uptake, restricted Na^+^ transport to shoots, vacuolar sequestration of excess Na^+^, and sustained uptake of potassium (K^+^) [21,22,23,24,25,26,27]. Potassium is an essential macronutrient involved in enzyme activation, protein synthesis, osmotic regulation, and stomatal function [28,29]. Under saline conditions, high Na^+^ concentrations compete with K^+^ uptake, leading to K^+^ deficiency and metabolic dysfunction [30,31,32]. Maintaining a higher K^+^/Na^+^ ratio is therefore widely recognized as a key indicator of salinity tolerance [33,34]. Moreover, K^+^ plays an important role in regulating Na^+^ influx through non-selective ion channels, thereby mitigating the detrimental effects of salt stress [35].

Salinity stress also significantly affects photosynthetic performance and plant growth, particularly during early vegetative stages. Excess Na^+^ accumulation disrupts ionic balance and reduces photosynthetic efficiency by altering chlorophyll stability and stomatal conductance [36,37]. High salinity has been reported to increase canopy temperature and significantly reduce plant height, leaf area, shoot biomass, and root dry weight [37]. Additionally, salt stress impairs starch metabolism, respiration, and hormonal balance, leading to reduced accumulation of photosynthetic pigments and carotenoids [38,39]. Maintenance of chlorophyll and carotenoid content under saline conditions is therefore considered an important adaptive response associated with improved stress resilience.

Screening for salinity tolerance under heterogeneous field conditions is challenging due to spatial variability in soil salinity and environmental fluctuations. Controlled screening systems such as lysimeters and microplots provide a reliable alternative for evaluating plant responses under defined salinity levels. Lysimeters allow precise regulation of irrigation, measurement of plant–water–salt interactions, and assessment of solute transport dynamics, whereas microplots simulate semi-field conditions with controlled salt application. These systems facilitate accurate phenotyping of morpho-physiological and ionic traits and enable efficient identification of tolerant genotypes at early growth stages [23].

Although numerous studies have evaluated morphological traits, ion accumulation, and photosynthetic parameters under salinity stress, these components are often studied independently. Limited information is available regarding their integrative assessment to elucidate distinct tolerance strategies in maize at early growth stages. In particular, the relationship between photosynthetic pigment stability and ion homeostasis mechanisms under graded salinity conditions remains insufficiently explored. A comprehensive evaluation of seedling growth responses, ionic balance, and pigment dynamics under controlled salinity gradients is therefore essential to identify reliable selection criteria for breeding salt-tolerant maize cultivars.

Therefore, the present study aimed to (i) quantify the effects of salinity on seedling growth, physiological attributes, and vigor of maize, and (ii) evaluate variations in photosynthetic pigments and major mineral composition across salinity gradients to identify key adaptive traits associated with salinity tolerance. The findings are expected to contribute to improved phenotyping strategies and the development of stress-resilient maize genotypes suitable for salt-affected environments.

## 2. Results

### 2.1. Genotypic Variation and Treatment Effects Under Salinity Stress

Maize genotypes were grown in microplots and lysimeters maintained at the seedling stage to evaluate the effects of different salinity levels on early plant growth. Across treatments (T1: dS/m3 dS/m; T2: dS/m6 dS/m; T3: dS/m9 dS/m), key physiological and growth traits like chlorophyll a (Chl a), chlorophyll b (Chl b), total chlorophyll, carotenoids, shoot fresh weight (SFW) and shoot dry weight (SDW) and plant height (PH) were recorded to assess the response of maize to salinity stress. The analysis of variance (ANOVA) revealed significant genetic variability among the genotypes for most traits. Genotype, treatment, and genotype × treatment (G × T) effects were highly significant (*p* < 0.001) for SFW, SDW, and PH, while Chl b was significant at *p* < 0.01 for genotype, while treatment was significant at *p* < 0.5. In contrast, Chl a, total chlorophyll, and carotenoids showed non-significant effects for genotype, treatment, and G × T, and the G × T effect for Chl b was also non-significant. Finally, the ANOVA confirmed that interaction effects were significant for most traits, as presented in Table 1.

A post hoc Tukey’s honestly significant difference (HSD) test was also analyzed to determine the appropriate confidence level to minimize the error. Effect of levels of salinity stress treatments on photosynthetic pigments (Chl a, Chl b, total chlorophyll, and carotenoids), SFW, SDW, and PH in maize genotypes using Tukey’s test. Different letters on trait mean values within columns indicate significant differences at *p* < 0.05. The significant factors were the control, treatment 1 (T1: dS/m3 dS/m), treatment 2 (T2: dS/m6 dS/m), and treatment 3 (T3: dS/m9 dS/m) in relation to Chl b, SFW, SDW, and PH. In contrast, Chl a, total chlorophyll, and carotenoids showed non-significant results (Table 2). Shoot fresh weight (SFW) declined by 50.69%, 67.43%, and 82.20% under T1, T2, and T3, respectively; SDW showed a similar trend, decreasing by 49.87%, 66.71%, and 82.01% at the corresponding stress levels. Similarly, PH was comparatively less affected, but still exhibited notable reductions of 12.97%, 33.02%, and 55.59% under T1, T2, and T3, respectively.

### 2.2. Physio-Morphological Responses of Maize Under Salinity Stresses

**Figure 1:**
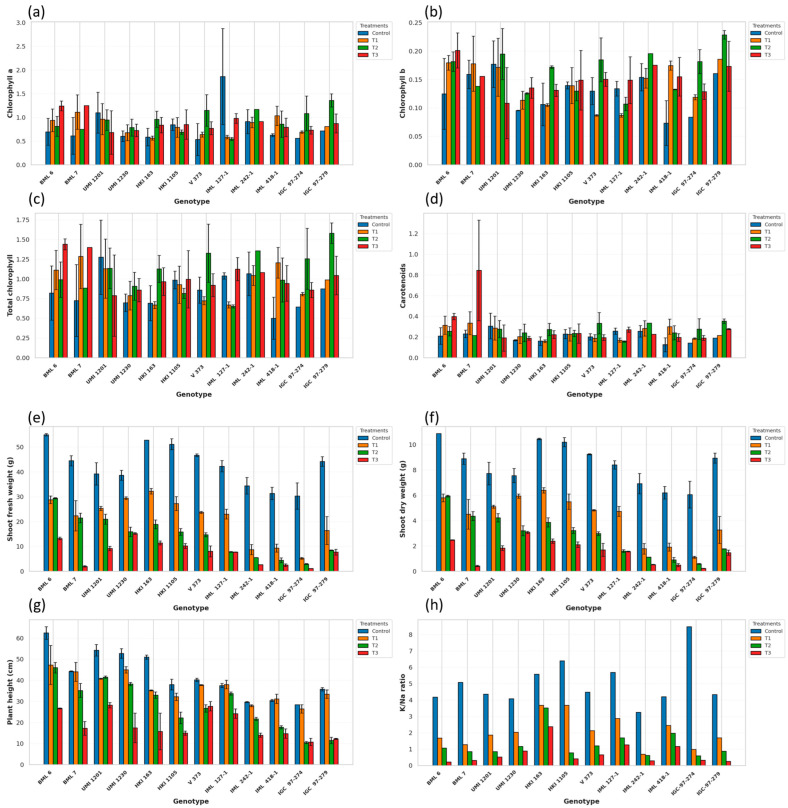
(**a**–**h**) Effect of different levels of salt concentrations (T1: dS/m3 dS/m, T: dS/m6 dS/m and T3: dS/m9 dS/m) on different traits at seedling stage of 12 maize genotypes: (**a**) chlorophyll a, (**b**) chlorophyll b, (**c**) total chlorophyll, (**d**) carotenoids, (**e**) shoot fresh weight, (**f**) shoot dry weight, (**g**) plant height, and (**h**) K^+^/Na^+^ ratio. The bars represent mean ± SE (n = 3).

Salt stress significantly affects plant growth and morphological attributes in maize. Although photosynthetic pigments did not show significant differences among treatments, numerical variations were observed. Table 1 indicates that chlorophyll a content is not significantly different across the genotypes in different treatments (control, T1, T2, T3), but there were slight increases in some specific genotypes. The genotypes BML 6 and BML 7 maintained comparatively higher Chl a in the high-salinity control (T3), indicating the resilience of the photosynthetic pigment under salt stress (Figure 1a). Additionally, IML 127-1 showed the highest Chl a content under the stress-free or control condition and a severe reduction under the highest-salinity treatment (T3), reflecting strong photosynthetic capacity under non-stress conditions. Overall, it indicated that Chl a is less sensitive to salt stress.

The Chl b content also exhibited noticeable genotypic variation and differential sensitivity to salinity stress across treatments. Relatively higher Chl b levels were observed in IGC-97-279 and IML 242-1 under T2 and BML 7 in T3, whereas UMI 1230 recorded comparatively lower values. The decline was particularly pronounced in UMI 1201, reflecting their greater susceptibility to salt stress. In contrast, BML 7, IGC-97-279, and IML 242-1 maintained relatively higher chlorophyll b levels across salinity treatments (Figure 1b). The total chlorophyll content varied significantly among the maize genotypes and declined progressively with increasing salinity stress. Under control conditions, the highest total chlorophyll levels were recorded in IGC-97-279, BML 7 and IML 242-1, whereas comparatively lower values were observed in UMI 1230, HKI 1105 and V 373. Genotypes such as IML 418-1, UMI 1230 and V 373 showed sharper reductions, indicating greater sensitivity to salinity. In contrast, BML 6, BML 7, IGC-97-279 and IML 242-1 retained relatively higher total chlorophyll (Figure 1c). The carotenoid content exhibited noticeable genotypic variation and showed differential responses to salinity stress across the treatments. Under salinity stress, a marked increase in carotenoid content was particularly evident in BML 7 at T3, followed by IGC-97-279 and IML 242-1 in T2, indicating a strong stress-induced carotenoid accumulation. In contrast, relatively smaller changes in carotenoid levels across the salinity treatments were observed in HKI 163 and IML 418-1, suggesting a limited carotenoid induction under stress conditions (Figure 1d). Among the 12 evaluated genotypes, BML 7, IGC-97-279 and IML 242-1 maintained comparatively higher levels of photosynthetic pigments such as Chl a, Chl b, total chlorophyll and carotenoid contents under stress conditions (T1, T2 and T3), indicating greater pigment stability under salinity. In contrast, UMI 1230, V 373, HKI 163, and IML 418-1 consistently recorded lower Chl a, Chl b, total chlorophyll and carotenoid contents, reflecting their higher sensitivity to salinity stress.

There were significant differences in growth-related parameters like shoot fresh weight, shoot dry weight and plant height (Figure 1e–g). Shoot fresh weight exhibited a strong, progressive, and statistically significant reduction with increasing salinity across all genotypes (Control > T1 > T2 > T3). Under control conditions, BML 6 recorded the highest SFW (~57 g), followed by HKI 163 and HKI 1105 (~53 g), while comparatively lower values were observed in IML 418-1, IGC-97-274 and IGC-97-279. With the imposition of salinity stress (T1: dS/m3 dS/m to T3: dS/m9 dS/m), all genotypes exhibited a sharp and consistent reduction in shoot fresh biomass, with the greatest decline occurring under the severe stress level (T3: dS/m9 dS/m). The reduction was particularly drastic in BML 7, IML 418-1, UMI 1201 and IGC-97-274, indicating higher sensitivity to salinity. In contrast, BML 6, HKI 1105 and UMI 1230 retained relatively higher shoot fresh weight across stress treatments, suggesting better tissue hydration and growth stability under saline conditions (Figure 1e). The SDW exhibited substantial genotypic variation and showed a marked decline with increasing salinity levels across the treatments. Under control conditions, the highest SDW was recorded in BML 6, HKI 1105 and V 373, whereas comparatively lower values were observed in IML 418-1 and IGC-97-274. Salinity stress caused a progressive reduction in shoot biomass from T1 (3 dS/m) to T3 (dS/m6 dS/m) in all genotypes, with the most severe decline occurring under T3 (9 dS/m). The reduction was particularly pronounced in IML 418-1, IGC-97-274 and UMI 1201, indicating greater sensitivity to salt stress. In contrast, BML 6, HKI 1105 and V 373 retained relatively higher SDW across the stress treatments. Across all salinity treatments, among all the genotypes, BML 6, HKI 1105 and V 373 consistently maintained higher SFW and SDW, reflecting better growth stability under saline conditions, whereas IML 418-1, IGC-97-274 and IGC-97-279 exhibited a pronounced reduction in both SFW and SDW, remaining the poorest performers under salinity stress.

Plant height declined progressively with increasing salinity stress across all the salinity treatments. Under control conditions, the tallest plants were recorded in BML 6, UMI 1201, UMI 1230 and HKI 163, whereas comparatively shorter plants were observed in IML 418-1, IML 242-1 and IGC-97-274. With the imposition of salinity stress (T1, T2 and T3), all genotypes exhibited a gradual reduction in plant height, and the magnitude of reduction was most pronounced under the severe stress level (T3). The decline was particularly sharp in UMI 1201, IML 418-1 and IGC-97-274, indicating greater sensitivity to salinity. In contrast, the groups that showed the least reduction or maintained their height across treatments in comparison to the control were V 373 and IML 127-1 (Figure 1g).

The K^+^/Na^+^ ratio varied markedly among the maize genotypes and declined sharply with increasing salinity stress across the treatments. Under control conditions, the highest K^+^/Na^+^ ratios were recorded in IGC-97-274, HKI 1105 and HKI 163, whereas comparatively lower values were observed in UMI 1201, IML 418-1 and IGC-97-279. With the imposition of salinity stress (T1, T2 and T3), a progressive decline in the K^+^/Na^+^ ratio was observed in all genotypes, indicating increased Na^+^ accumulation and impaired ionic balance under higher salt levels. The reduction was most pronounced under T3, particularly in UMI 1201, IML 418-1 and IGC-97-274, suggesting greater ionic stress sensitivity. In contrast, HKI 163 maintained comparatively higher K^+^/Na^+^ ratios across stress treatments, reflecting their superior ability to restrict Na^+^ buildup and retain K^+^ homeostasis under saline conditions (Figure 1h). Moreover, based on the bar graph of significant traits, BML 6 and UMI 1230 showed the least reduction over the treatment and can be considered as tolerant, while IGC-97-274 and IGC-97-279 can be considered as sensitive, as they showed greater reductions. To differentiate tolerant and sensitive genotypes, the cut-off values for physiological traits were defined based on their relative reduction under salinity stress compared with the control and their grouping in the multivariate analysis. Genotypes exhibiting lower reductions in chlorophyll b and carotenoid contents and maintaining higher K^+^/Na^+^ ratios under stress were categorized as tolerant, whereas those showing greater pigment degradation and lower ionic ratios were considered sensitive. This classification was consistent with the clustering pattern obtained from principal component analysis and stress tolerance indices.

### 2.3. Evaluation of Maize Genotype Under Salinity Treatments Using Stress Indices

Across all salinity levels, the genotypes exhibited distinct and consistent patterns of variation in total chlorophyll retention and stress tolerance indices. Stress tolerance was evaluated using mean productivity (MP), tolerance index (TOL), yield stability index (YSI), harmonic mean (HM), stress tolerance index (STI), mean yield under stress (Ys), mean yield under non-stress (Yp), mean yield (M) and stress susceptibility index (SSI). Under mild salinity levels (T1: dS/m3 dS/m), genotypes such as IML 418-1, BML 7 and BML 6 showed low TOL (TOL: −0.704, −0.293 and −0.56) and high YSI (YSI: 0.40, 1.35 and 1.77) values, indicating minimal stress-induced reduction and strong early-stage tolerance. At moderate salinity (T2: dS/m6 dS/m), IGC-97-279, IML 418-1, IGC-97-274 and IML 242-1 maintained comparatively higher stability, supported by high HM and STI values together with low SSI. Under severe salinity (T3: dS/m9 dS/m), BML 7, IML 418-1, IML 127-1 and IGC-97-279 exhibited markedly negative TOL values and elevated STI and YSI scores. Collectively, total chlorophyll in BML 7, IML 418-1, IGC-97-274, and IGC-97-279 under all three salinity stress regimes highlights their superior stability and tolerance (Table S1). Conversely, UMI 1201 exhibited a positive TOL index, reduced YSI, and the maximum chlorophyll loss, substantiating its classification as the most susceptible genotype. Carotenoid content showed clear and consistent differences among the genotypes across the three salinity levels. Under mild stress level (T1: dS/m3 dS/m), IML 242-1, BML 6, BML 7, and IML 418-1 showed negative or near-zero TOL values together with high YSI and moderate STI, indicating stress-induced enhancement of carotenoid content and effective early ionic adjustment. At moderate salinity (T2: dS/m6 dS/m), genotypes IGC-97-279, IGC-97-274, IML 418-1, V 373, and IML 242-1 maintained comparatively greater stability, reflected in negative TOL, high HM and STI, and lower SSI, suggesting efficient carotenoid retention and metabolic resilience, whereas under severe stress (T3: dS/m9 dS/m), genotypes BML 7 and BML 6 exhibited the strongest carotenoid amplification, supported by highly negative TOL and very high YSI and STI (Table S1). Taken together, the results indicate that BML 6, BML 7, and IML 242-1 represent the most carotenoid-stable and stress-responsive genotypes across all salinity intensities, whereas UMI 1201 and IML 418-1 are the least carotenoid-resilient genotypes under all salinity levels.

For SFW across all genotypes, the magnitude of reduction intensified across salinity levels (T1 to T2 and T3). Under control conditions, SFW ranged from 30.28 g (IGC-97-274) to 54.9 (BML 6) g, whereas under salinity stress, the values decreased sharply according to stress severity, with the greatest loss observed under T3. Across the stress treatments, UMI 1230, UMI 1201, BML 6, and HKI 163 consistently retained higher SFW, as reflected by lower TOL values together with higher HM, STI, and YSI, indicating superior tolerance and stability. UMI 1230 exhibited the lowest TOL and highest YSI under T1 and T3, while UMI 1201 maintained moderate but stable performance across stress levels. BML 6 recorded the highest HM and STI under T1 and T2 and sustained relatively better biomass under T3, similar to HKI 163. In contrast, IGC-97-274, IML 242-1, and IML 418-1 displayed high TOL and SSI with very low STI and YSI, indicating strong susceptibility. Overall, UMI 1230, BML 6, UMI 1201, and HKI 163 emerged as relatively stable and salt-tolerant genotypes (Table S1). Similarly, SDW, across all genotypes, showed a magnitude of decline that increased from T1 (3 dS/m) to T2 (6 dS/m) and T3 (9 dS/m). While most genotypes maintained moderate SDW under T1, a sharp reduction was observed under T2, and the most drastic loss occurred under T3, indicating severe growth inhibition at higher salinity intensity. Across stress levels, UMI 1230, UMI 1201, BML 6, and HKI 163 consistently retained higher SDW, as evidenced by lower TOL values together with higher HM, STI, and YSI, demonstrating greater stability and tolerance. UMI 1230 showed the lowest TOL and highest YSI under T1 and T3, whereas UMI 1201 maintained reliable performance across T1 and T2. BML 6 recorded the highest HM and STI under T2 and sustained comparatively better biomass under T3, similar to HKI 163. In contrast, IGC-97-274, IML 242-1, and IML 418-1 displayed high TOL and SSI with extremely low STI and YSI, indicating high susceptibility, particularly under T3. Therefore, based on SFW and SDW, the genotypes UMI 1230, BML 6, UMI 1201, and HKI 163 were identified as relatively salt-tolerant genotypes.

Plant height declined progressively from T1 to T3 under all salinity levels (Table S1). Under control conditions, plant height ranged from 62.5 cm (BML 6) to 28.5 cm (IGC-97-274), whereas under stress, it decreased to 47.25–26.75 cm in T1 (3 dS/m), 46.0–10.5 cm in T2 (6 dS/m), and 28.25–10.75 cm in T3 99 dS/m). Genotypes BML 6, UMI 1201 and UMI 1230 maintained comparatively greater height across environments, supported by consistently higher HM and STI values (HM = 53.82, 52.99, 37.46 and STI = 1.667, 1.623, 0.944 across T1–T3 for BML 6), indicating strong performance under both control and stress conditions, although their high TOL under T3 (9 dS/m) suggested sensitivity under severe stress. In contrast, IML 127-1 and V 373 showed low TOL and SSI together with higher YSI across stress levels, confirming greater stability and tolerance. Conversely, IGC-97-274 and IGC-97-279 exhibited very high TOL and SSI and extremely low HM, STI and YSI, identifying them as highly susceptible, particularly under T2 and T3. Overall, the combined analysis indicates that IML 127-1 and V 373 are the most stable and stress-tolerant genotypes, whereas IGC-97-274 and IGC-97-279 remain the most sensitive under increasing salinity intensity.

Across all stress levels (T1: dS/m3 dS/m, T2: dS/m6 dS/m, and T3: dS/m9 dS/m), a progressive decline in the K^+^/Na^+^ ratio was observed among the genotypes, with the reduction being most pronounced under T3. Under control conditions, the K^+^/Na^+^ ratio ranged from 3.26 (IML 242-1) to 8.48 (IGC-97-274), whereas under stress, it declined to 0.69–3.68 in T1, 0.60–3.53 in T2, and 0.22–2.38 in T3. Based on TOL values, IML 418-1 and HKI 163 exhibited the lowest reductions across stress intensities, indicating better ionic balance, while there was very high TOL in IGC-97-274 and HKI 1105, which reflected strong susceptibility. Harmonic mean values further supported the superiority of HKI 163 and IML 418-1, whereas IML 242-1 and BML 6 recorded the lowest stability under higher stress. SSI values indicated minimal susceptibility in HKI 163 and IML 418-1, in contrast to IGC-97-274 and BML 7, which showed consistently high sensitivity. Correspondingly, STI and YSI remained highest for HKI 163 and IML 418-1, confirming superior ionic stability and salt tolerance, while IGC-97-274 and IML 242-1 recorded the poorest performance (Table S1). Overall, HKI 163, IML 418-1 and IML 127-1 emerged as the most tolerant genotypes, whereas IGC-97-274, HKI 1105, BML 7 and IML 242-1 were highly sensitive to salinity.

Across all salinity stress levels and studied traits, a clear divergence was observed between consistently tolerant and highly sensitive genotypes. Genotypes BML 7, IML 127-1, HKI 163, UMI 1201 and V 373 constantly exhibited lower TOL, higher HM, STI and YSI values, together with lower SSI, indicating superior physiological stability, efficient ion regulation, enhanced pigment protection, and greater biomass retention under increasing salinity stress. In particular, HKI 163 and IML 127-1 showed strong ionic homeostasis and K^+^/Na^+^ stability; BML 7 and BML 6 demonstrated high chlorophyll and carotenoid stability, while UMI 1230, BML 6, UMI 1201 and HKI 163 consistently maintained higher shoot biomass and plant height across T1–T3, confirming their classification as the most salt-tolerant and stable genotypes overall. In contrast, genotypes IGC-97-274, IML 242-1, IML 418-1, IGC-97-279, HKI 1105 and UMI 1201 exhibited high TOL and SSI along with very low STI, HM and YSI, indicating severe pigment degradation, poor ionic regulation and drastic biomass loss under stress. Among these, IGC-97-274, IML 242-1, IGC-97-279 and HKI 1105 were identified as the most sensitive genotypes, showing maximum reduction and minimum stability across traits and stress intensities. Moreover, based on the composite salt tolerance index using the ranks of all the significant traits, BML 6, HKI 163, UMI 1230 and HKI 1105 showed comparable tolerance, and IML 418-1, IML 242-1, IGC-97-274 and IGC-97-279 were sensitive to salt.

### 2.4. Principal Component Analysis for Trait Contribution and Genotype Differentiation

**Figure 2:**
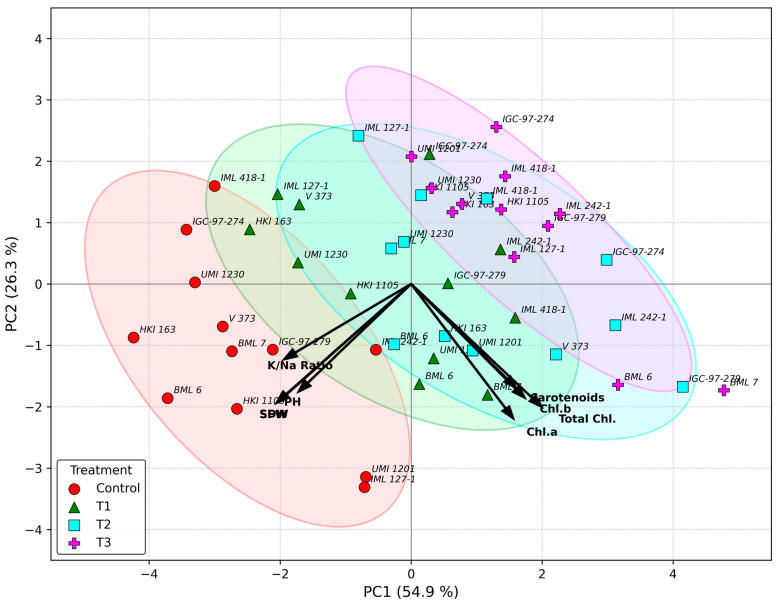
Principal component analysis (PCA) showing the relationships among 8 morpho-physiological and ionic traits and maize genotypes under salinity stress. PC1 and PC2 explained 54.9% and 26.3% of the total variation, respectively. Genotypes are represented by points, and trait vectors indicate their contribution to the principal components. Chl a—chlorophyll a; Chl b: chlorophyll b; total Chl: total chlorophyll; SFW: shoot fresh weight; SDW: shoot dry weight; PH: plant height; K^+^/Na^+^ ratio: potassium and sodium ratio.

The PCA biplot explained 54.9% of the total variation along PC1 and 26.3% along PC2, together accounting for 81.2% of the total variability among genotypes and traits under different salinity levels. Traits related to photosynthetic pigment (Chl a, Chl b, total chlorophyll and carotenoids) showed strong positive loadings on PC1 and clustered toward the right side of the biplot, indicating a strong association among these traits and their contribution to tolerance under higher salinity levels. Genotypes such as IML 418-1, IGC-97-274, IML 242-1, and V 373 under T3 conditions were positioned near these traits, suggesting better pigment retention and stress-responsive physiological adjustments under severe salinity. Conversely, SFW, SDW, PH, and K^+^/Na^+^ ratio were positioned toward the negative side of PC1 and lower PC2, indicating their stronger association with the control and T1 conditions (Figure 2). Genotypes, including BML 6, BML 7, UMI 1230 and HKI 163, that were grouped near these traits reflected higher biomass retention and ionic balance under mild stress and non-stress environments. Highly stress-tolerant genotypes, such as UMI 1230, HKI 163 and BML 6, under control conditions, were clearly separated from T3 clusters, confirming strong treatment discrimination.

## 3. Discussion

Salinity stress is one of the major abiotic constraints limiting maize crop productivity worldwide, as it adversely affects plant growth, physiological processes, and yield [40,41]. Under saline conditions, plants experience a dual stress environment characterized by ionic toxicity and osmotic imbalance, resulting from the simultaneous effects of excess salt ions and reduced water availability [42]. The present study focused on the evaluation of traits at the seedling stage, including chlorophyll a (Chl a), chlorophyll b (Chl b), total chlorophyll, carotenoids, shoot fresh weight (SFW), shoot dry weight (SDW), plant height (PH), and the K^+^/Na^+^ ratio. Pigment-based tolerance supports photosynthetic stability and is useful for early-stage screening under moderate salinity, whereas ion homeostasis-based tolerance ensures better Na^+^ exclusion, higher K^+^/Na^+^ ratio, and sustained yield under severe salinity. Combining both mechanisms offers a practical breeding strategy for developing widely adapted salt-tolerant maize cultivars. The objective of this study was to identify the most suitable trait for screening maize genotypes under salt stress.

The ANOVA results demonstrated that salinity had a strong and differential impact on maize seedlings, as reflected by significant genotype, treatment, and G × T interactions for SFW, SDW and PH. This indicates substantial genetic variation in growth resilience and genotype-specific plasticity under saline conditions, consistent with earlier reports in maize and other cereals [12,43], whereas biomass-related traits are more sensitive indicators of stress response [44,45]. Tukey’s HSD test further confirmed significant reductions in Chl b, SFW, SDW and PH with increasing salinity, reinforcing the suppressive effects of salt stress on cell expansion, carbon assimilation and structural growth [37]. Similar trends reported in previous studies support the suitability of biomass and morphological traits as reliable selection criteria for screening salt-tolerant maize genotypes at early growth stages [46,47].

Genotypes such as BML 7, IGC-97-279 and IML 242-1 exhibited relatively higher retention of Chl a, Chl b, total chlorophyll and carotenoid content under stress. Although these traits showed limited statistical significance among genotypes, their stability may still reflect supportive physiological adjustments that help maintain photosynthetic activity under saline conditions. Although chlorophyll content showed some genotypic variation under salinity stress, its response was comparatively less consistent and less strongly associated with biomass and ionic balance than other measured traits. This suggests that chlorophyll content alone may not be a reliable selection criterion for salinity tolerance in maize; however, it remains a supportive physiological indicator when used in combination with growth and ion-homeostasis traits [48]. Chlorophyll content shows a strong positive correlation with plant nitrogen levels; higher nitrogen increases leaf chlorophyll concentration and enhances photosynthetic capacity, as nitrogen is a key component of the chlorophyll molecule [49].

Growth-related parameters such as plant height, shoot fresh weight (SFW), and shoot dry weight (SDW) are widely used indicators of plant vigor and stress tolerance. In the present study, these traits declined progressively with increasing salinity levels, reflecting the combined effects of osmotic stress and ion toxicity on plant growth. High salt concentrations reduce soil water potential, limiting water uptake and restricting cell expansion and metabolic processes necessary for plant development. Consequently, biomass reduction under saline conditions can be attributed to osmotic stress during early exposure and ionic toxicity at later stages [50]. Despite this general decline, genotypic differences were evident in the ability to maintain growth under saline conditions. Genotypes such as BML 6, HKI 1105, and HKI 163 showed comparatively smaller reductions in SFW, SDW, and plant height, suggesting better osmotic adjustment and physiological stability. Similar observations have been reported in maize, where tolerant genotypes maintained higher shoot biomass and plant height under salinity stress [49]. In contrast, genotypes such as IML 418-1, IGC-97-274, and IGC-97-279 exhibited greater reductions in growth traits, indicating higher sensitivity to salinity stress. These reductions may result from impaired water uptake, disrupted nutrient transport, and metabolic imbalance caused by excessive salt accumulation. Comparable decreases in plant height and shoot biomass under salinity stress have also been reported in maize and other cereal crops [51,52]. Tolerant genotypes such as HKI 163, HKI 1105, and IML 127-1 maintained comparatively higher K^+^/Na^+^ ratios under saline conditions, suggesting efficient mechanisms of sodium exclusion and potassium retention. These mechanisms may include selective ion transport at the root level, compartmentalization of Na^+^ into vacuoles, and enhanced activity of membrane transport proteins involved in ion regulation. Previous studies have demonstrated that the maintenance of a favorable K^+^/Na^+^ ratio is a critical component of salinity tolerance in plants because it helps preserve cellular homeostasis and metabolic activity under stress conditions [12,13,24,53]. In contrast, sensitive genotypes such as UMI-1201 and IML-418-1 showed a sharp decline in the K^+^/Na^+^ ratio, indicating poor regulation of sodium uptake and greater susceptibility to ionic toxicity. Excessive sodium accumulation can lead to membrane depolarization and potassium leakage, ultimately causing metabolic imbalance and growth inhibition. Similar patterns of ionic imbalance have been reported in sensitive maize [54] and wheat genotypes exposed to salinity stress [47,55].

To further evaluate genotypic performance under saline environments, several stress tolerance indices were calculated. These indices provide a quantitative framework for comparing genotype performance under stress and non-stress conditions. In the present study, indices such as the stress tolerance index (STI), yield stability index (YSI), and harmonic mean (HM) were positively associated with biomass maintenance under salinity, while the stress susceptibility index (SSI) and tolerance index (TOL) reflected the degree of yield reduction under stress. Genotypes such as BML 6, HKI 163, UMI 1230, and HKI 1105 showed relatively higher STI, YSI, and HM values together with lower SSI and TOL values, indicating better stability and adaptability under saline conditions. These results support the classification of these genotypes as relatively tolerant, as they were able to maintain higher biomass and physiological stability across stress levels. The integration of multiple stress indices provided a more robust basis for genotype classification than relying on individual traits alone. Genotypes with higher STI and YSI values maintained better productivity under stress conditions, while lower SSI and TOL values indicated reduced sensitivity to salinity. The close association between favorable stress index values and higher K^+^/Na^+^ ratios further confirms that ionic balance plays a central role in determining genotypic responses to salinity stress [24,53,56]. In contrast, genotypes such as IGC-97-274 and IML 242-1 showed higher SSI and TOL values, reflecting greater susceptibility and larger reductions in biomass under saline conditions. Similar genotypic differences in sodium accumulation and salt tolerance have been reported in maize cultivars and other crop species [57,58,59,60]. Comparable mechanisms of ion regulation and stress tolerance have also been observed in plants such as Solanum scabrum [61], rice [62], and flax [63].

The PCA provided functional interpretation of the multivariate data. PC1, which explained the largest share of variation, was positively associated with biomass-related traits, plant height, chlorophyll, carotenoids, and the K^+^/Na^+^ ratio, and negatively associated with Na^+^ content, indicating that this component represents overall salinity tolerance driven by maintenance of photosynthetic efficiency and ionic homeostasis. PC2 mainly reflected growth reduction and ion toxicity effects under increasing salinity. The biplot clearly separated tolerant and sensitive genotypes. BML 6, UMI 1230 and HKI 163 were positioned along the positive side of PC1, reflecting their ability to maintain higher growth, pigment stability, and a favorable K^+^/Na^+^ ratio, whereas sensitive genotypes were associated with higher Na^+^ accumulation and reduced growth.

Similar associations between pigment stability, oxidative stress mitigation, and salinity tolerance have been reported in maize and other cereals [57,64,65,66]. In contrast, SFW, SDW, PH and K^+^/Na^+^ ratio clustered toward the negative axis of PC1, together with genotypes such as BML 6, BML 7, UMI 1230 and HKI 163, indicating stronger relationships with performance under control and mild stress (T1). These genotypes therefore exhibited tolerance mechanisms driven primarily by biomass maintenance, ionic balance and growth stability, which are widely recognized as key features of salt-tolerant maize lines at early growth stages [43,47]. The clear separation of T3 clusters from control-associated genotypes further confirms that PCA effectively discriminated between tolerant versus sensitive response strategies under varying salinity intensities. The strong association of biomass traits (SFW, SDW, and plant height) together with the K^+^/Na^+^ ratio on PC1 indicates that maintenance of growth and ionic balance represents the primary physiological driver of salinity tolerance among the evaluated genotypes. PCA results emphasize that two complementary tolerance strategies operate among maize genotypes under salinity: (i) pigment-stability-based stress acclimation under severe stress, and (ii) biomass- and ion-homeostasis-based tolerance under mild–moderate stress. Integration of both strategies has been suggested as a desirable target for improving salt resilience in maize breeding programs [12,53].

## 4. Materials and Methods

### 4.1. Plant Materials and Experimental Details

A total of 12 maize genotypes, including advanced breeding lines, wild species and parental lines of popular released hybrids (Table 3), were evaluated in a randomized complete block design (RCBD) with three replications. The salinity experiment was conducted during the 2020–2021 *Rabi* season at the Indian Council of Agricultural Research (ICAR)–Central Soil Salinity Research Institute (CSSRI), Regional Research Station, Bharuch, Gujarat, India (23°54′ N, 74°57′ E), under semi-arid climatic conditions. The study was initially established at the seedling stage using microplots and lysimeters to precisely control salinity levels and soil moisture regimes. The experiment comprised three salinity treatments, T1 (3 dS/m), T2 (6 dS/m), and T3 (9 dS/m), along with a non-saline control. The 6 plants/row in the microplot and 2 plants/line in the lysimeter were planted with a recommended spacing of 60 × 25 cm. Salinity stress was imposed through the application of naturally saline irrigation water with an electrical conductivity of irrigation water (ECiw) of approximately 3, 6, and 9 dS/m for T1, T2, and T3, respectively, whereas the control plots received good-quality water with an ECiw of about 0.30 dS/m. Salinity levels were maintained consistently throughout the crop growth period. The experimental soil was clay loam in texture, with a pH ranging from 7.01 to 8.53 and an electrical conductivity of the saturated extract (ECe) between 6.13 and 9.58 dS/m. Other soil physicochemical properties recorded during the crop season included: exchangeable sodium percentage (ESP) of 2.39–8.02%, sand content of 24.6–27.6%, silt 18.3–28.3%, clay 45.2–56.1%, organic carbon content of 0.43%, available nitrogen ranging from 160 to 190 kg ha^−1^, Olsen phosphorus from 11 to 26 kg ha^−1^, and available potassium between 682 and 840 kg ha^−1^. In the lysimeter and microplot experiments, irrigation water of four salinity levels (Control: 0.3; T1: 3; T2: 6; and T3: 9 dS/m) was prepared with defined cation–anion compositions. The saline treatments showed a progressive increase in Na^+^ and Cl^−^ concentrations from T1 to T3, while Ca^2+^ and Mg^2+^ remained relatively stable, with moderate variation in CO_3_^2−^ and HCO_3_^−^ levels (Table 4).

### 4.2. Measurement of Physio-Morphological Traits

Morphological traits of all the genotypes were measured at the seedling stage. Three plants of every genotype in every replicate were used to record the data on chlorophyll a (Chl a), chlorophyll b (Chl b), total chlorophyll (total Chl), carotenoids, shoot fresh weight (SFW) in g, shoot dry weight (SDW) in g, plant height (PH) in cm and K^+^/Na^+^ ratio under the salinity and non-salinity (control) conditions. Each seedling was cut down at the junction of the shoot to separate each part. Shoot fresh and dry weights were measured using a digital electronic balance (PAJ1003, OHAUS, Parsippany, NJ, USA), and the samples were dried in an oven at 65 °C for 4–5 days to record the shoot dry weight.

### 4.3. Estimation of Chlorophyll and Carotenoid Content

Chlorophyll and carotenoid content were estimated as per the method described by [67]. The procedure for estimation of chlorophyll content in plants is based on the absorption of light by chlorophyll extracts prepared by incubating the leaf tissues in DMSO (dimethyl sulfoxide). DMSO renders plasmalemma permeable, thereby causing the leaching of the pigments [67]. The absorbance of the known volume of solution containing a known quantity of leaf tissue at two respective wavelengths (663 and 645) was determined for the chlorophyll content and at 480 nm for the total carotenoid content. Chlorophyll a, chlorophyll b and total chlorophyll content were estimated using the formula given by Arnon and Whatley 1949 [68], while carotenoid content was determined by following the formula given by Lichtenthaler and Welburn 1983 [69]. A total of 30 mg fresh leaf samples were added to the test tubes containing 4 mL DMSO. Tubes were kept in the dark for 4 h at 65 °C. Then the samples were taken out and cooled at room temperature, and the absorbance was recorded at 663, 645 and 480 nm using DMSO as a blank and was expressed as mg g^−1^ fresh weight. The following formulas were used for the calculation of chlorophyll content and carotenoid content.

chlorophyll ‘a’ = (12.7 × A_663_ − 2.69 × A_645_) × V/1000 × w

chlorophyll ‘b’ = (22.9 × A_645_ − 4.68 x A_663_) × V/1000 × w

Total chlorophyll = (20.2 × A_645_ + 8.02 × A_663_) × V/1000 × w

Total carotenoids = (A_480_ + (0.114 × A_663_) − (0.638 × A_645_)) × V/(1000 × w)

where

A_663_ = Absorbance values at 663 nm;

A_645_ = Absorbance values at 645 nm;

A_480_ = Absorbance values at 480 nm;

W = Weight of the sample in g;

V = Volume of the solvent used (mL).

### 4.4. Estimation of Tissue Sodium (Na^+^), Potassium (K^+^) Concentration

The Na^+^ and K^+^ concentrations in the roots, lower leaves and upper leaf tissues of all the genotypes under the control and salinity stress conditions were estimated by a modified digestion protocol from Munns et al. 2010 [70]. We compared the modified digestion method with standard diacid digestion and got comparable results. Sampling was done at the seedling stage. Plant samples were oven-dried, and 100 mg of each dried sample was finely ground using a homogeniser and acid-digested using 10 mL of 0.5 N HNO_3_ and kept overnight on a shaker. The next day, the extract was kept in an oven at 80 °C for one hour. The extracts were then filtered through Whatman 40 grade filter paper. The Na^+^ and K^+^ concentrations in the extract were measured using a Flame Photometer (Ahmedabad, India).

### 4.5. Statistical Analysis

The analysis of variance was performed to determine whether significant differences existed among the means of all measured traits. When ANOVA indicated significant differences, Tukey’s honestly significant difference (HSD) test was applied as a post hoc procedure to compare pairwise treatment means. The principal component analysis (PCA) was employed to examine the multivariate structure of the dataset. This approach enabled the visualization of relationships among traits and treatments, helping to detect underlying patterns and trait grouping. All statistical analyses were conducted in R version 6.3.6. ANOVA, and post hoc tests were performed using *car* [71], while PCA was conducted using the *FactoMineR* package [72]. Graphs and visualizations were generated using the *ggplot2* package [73].

Salinity tolerance indices were calculated for all measured traits across both years using the online iPASTIC tool (https://manzik.com/ipastic/ (accessed on 10 January 2026)) [74] based on yield under non-stress (Yp) and salinity stress (Ys) conditions. Yield stability index (YSI), stress tolerance index (STI), stress susceptibility index (SSI), tolerance index (TOL), and harmonic mean (HM) were computed to assess genotypic performance across contrasting environments. These indices were used to screen maize genotypes for salinity tolerance, classifying genotypes with higher Ys and favorable index values as tolerant, while those with greater yield reductions (high TOL and SSI) were identified as sensitive. The different indices were calculated with the formulas given below as per the following researchers: SSI by Fischer & Maurer (1978) [75], TOL by Rosielle & Hamblin (1981) [76], STI by Fernandez (1992) [77], and YSI and HM by Bouslama & Schapaugh (1984) [78]:

Tolerance index (TOL)

TOL = Yp − Ys(1)

Stress susceptibility index (SSI)

SSI = [1 − (Ys/Yp)]/SI(2)

Stress intensity (SI)

SI = 1 − (Ȳs/Ȳp)(3)

Stress tolerance index (STI)

STI = (Yp × Ys)/(Ȳp^2^) (4)

Harmonic mean (HM)

HM = (2 × Yp × Ys)/(Yp + Ys)(5)

Yield stability index (YSI)

YSI = Ys/Yp(6)

where

Yp = Yield under non-stress (control) condition;

Ys = Yield under salinity stress condition;

Ȳp = Mean yield of all genotypes under control;

Ȳs = Mean yield of all genotypes under salinity stress;

SI = Stress intensity.

## 5. Conclusions

The present study revealed substantial genetic variability in salinity tolerance among the evaluated maize genotypes. Genotypes such as BML 6 and HKI 163 consistently exhibited superior performance across key morpho-physiological and ionic traits under both moderate and severe salinity levels. Their improved tolerance was associated with better maintenance of biomass, plant height, chlorophyll b and, most importantly, a higher K^+^/Na^+^ ratio, indicating efficient ionic homeostasis and sustained metabolic activity under stress conditions. In contrast, susceptible genotypes showed pronounced reductions in growth and physiological traits along with greater ionic imbalance, reflecting their limited adaptive capacity under salinity.

The results demonstrate that salinity tolerance in maize is governed by coordinated physiological mechanisms, in which the maintenance of photosynthetic pigments supports stress adaptation but operates in conjunction with effective ion regulation and growth stability. Among the studied traits, the K^+^/Na^+^ ratio emerged as a key determinant of tolerance, highlighting its potential as a reliable selection criterion for screening salt-tolerant genotypes at early growth stages. The identified genotypes also represent valuable genetic resources for breeding programs aimed at developing high-yielding maize cultivars with enhanced and stable salinity tolerance.

## Figures and Tables

**Table 1:** Analysis of variance for the effect of salinity stress on photosynthetic pigments, shoot fresh weight, shoot dry weight and plant height for 12 maize genotypes. The values given for each trait are mean sums of squares (MSSs).

Source of Variation	df	Chl a	Chl b	Total Chl	Carotenoids	SFW (g)	SDW (g)	PH (cm)
Genotype	11	0.082 ^ns^	0.004 **	0.106 ^ns^	0.028 ^ns^	366.852 ***	14.499 ***	537.076 ***
Treatment	3	0.086 ^ns^	0.006 *	0.261 ^ns^	0.029 ^ns^	5558.540 ***	217.464 ***	2506.896 ***
G × T	33	0.146 ^ns^	0.002 ^ns^	0.100 ^ns^	0.020 ^ns^	33.576 ***	1.394 ***	46.725 ***
Residuals (Error)	48	0.128	0.001	0.105	0.017	8.657	0.347	14.698

Significance: *** *p* < 0.001; ** *p* < 0.01; * *p* < 0.05; ns = non-significant, df; degree of freedom. Chl a = chlorophyll a; Chl b = chlorophyll b; Total Chl = total chlorophyll; SFW = shoot fresh weight; SDW = shoot dry weight; PH = plant height.

**Table 2:** Effect of levels of salinity stress treatments on photosynthetic pigments, shoot fresh weight, shoot dry weight and plant height in 12 maize genotypes using Tukey’s test. Different letters on trait mean values within columns indicate significant differences at *p* < 0.05. Trait values without any letter indicate non-significance.

Subject	Chl a	Chl b	Total Chl	Carotenoids	SFW (g)	SDW (g)	PH (cm)
Control	0.804	0.128 a	0.848	0.206	42.533 a	8.454 a	42.083 a
T1 (3 dS/m)	0.807	0.141 a	0.946	0.238	20.974 b	4.238 b	36.625 a
T2 (6 dS/m)	0.925	0.164 b	1.085	0.265	13.854 c	2.814 c	28.188 b
T3 (9 dS/m)	0.887	0.151 a	1.035	0.286	7.572 d	1.521 d	18.688 c
HSD Value	0.272	0.032	0.243	0.105	5.872	1.174	7.112

Chl a: chlorophyll a; Chl b: chlorophyll b; SFW: shoot fresh weight; SDW: shoot dry weight; PH: plant height; T1, T2, T3: treatment levels.

**Table 3:** Maize genotypes used for salinity screening at seedling stage.

S. No.	Genotypes	Pedigree	Source
1	BML 6	SRRL 65-B96-1-1-2- #-2-2-1-Ä-1-1-Ä^b^-Ä^b^	AICRP, Hyderabad
2	BML 7	[X_2_Y Pool × CML 226]-B 98 R-1-1-1-Ä^b^-Ä^b^-Ä^b^-Ä^b^-Ä^b^-Ä^b^	AICRP, Hyderabad
3	UMI 1201	Selection from W2625-3	AICRP, Coimbatore
4	UMI-1230	Selection from W2619-3	AICRP, Coimbatore
5	HKI-163	CML 163	AICRP, Karnal
6	HKI 1105	Cg-633	AICRP, Karnal
7	V 373	JKMH-175-4 (O.P.) ⊗-16-7-12-1	ICAR-VPKAS, Almora
8	IML 127-1	Waxy 418	ICAR-IIMR, Ludhiana
9	IML 242-1	MRCHY 5133-2-11-3-4	ICAR-IIMR, Ludhiana
10	IML 418-1	waxy 418	ICAR-IIMR, Ludhiana
11	IGC-97-274	*Zea mays* ssp. *mexicana*	ICAR-NIASM, Chhattisgarh
12	IGC-97-279	*Zea mays* ssp. *mexicana*	ICAR-NIASM, Chhattisgarh

**Table 4:** Cation–anion composition of various irrigation water treatments in lysimeter and microplots.

	mEq/L						
Treatment	Na	K	Ca	Mg	CO_3_^2−^	HCO_3_^−^	Cl
Control (0.3 dS/m)	0.67	0.04	1.4	1.4	0.0	2.8	3
T1 (3 dS/m)	18.81	0.03	1.2	4.6	1.8	7.3	24
T2 (6 dS/m)	24.44	0.04	1.4	4.6	1.2	6.9	48
T3 (9 dS/m)	38.03	0.05	1.6	4.0	1.4	5.9	67

## Data Availability

The original contributions presented in this study are included in the article/Supplementary Materials. Further inquiries can be directed to the corresponding author.

## Supplementary Materials

The following supporting information can be downloaded at https://www.mdpi.com/article/10.3390/ijms27073037/s1.

## References

[1] Ranum P., Peña-Rosas J.P., Garcia-Casal M.N. (2014). Global maize production, utilization, and consumption. Ann. N. Y. Acad. Sci..

[2] Sajid M., Rahim F., Ullah S., Ullah R., Bilqees R., Shakir L. (2023). Qualitative and quantitative ethnobotanical study of Arrang Valley of District Bajaur, Khyber Pakhtunkhwa, Pakistan. J. Agric. For. Res..

[3] Alka A.J., Singh M.K., Kumar S., Shambhavi S., Sneha (2020). Effect of plant geometry, graded fertility and zinc level on growth, yield and quality of baby corn fodder. Int. J. Chem. Stud..

[4] Shrivastava P., Kumar R. (2015). Soil salinity: A serious environmental issue and plant growth-promoting bacteria as a mitigation tool. Saudi J. Biol. Sci..

[5] Jat S.L., Jat H.S., Rakshit S., Sharma P.R., Kumar B., Kakraliya M., Gathala M.K., Bijarniya D., Kalwania K.C., Singh Y. (2025). Maize as an alternative to resource-intensive rice: Empirical insights from on-farm participatory study under diverse agricultural scenarios in the Indo-Gangetic Plains of Northwestern India. Front. Sustain. Food Syst..

[6] Das T.K., Saharawat Y.S., Bhattacharyya R., Sudhishri S., Bandyopadhyay K.K., Sharma A.R., Jat M.L. (2018). Conservation agriculture effects on crop and water productivity, profitability, and soil organic carbon accumulation under a maize–wheat cropping system in the north-western Indo-Gangetic Plains. Field Crops Res..

[7] Grote U., Fasse A., Nguyen T.T., Erenstein O. (2021). Food security and the dynamics of wheat and maize value chains in Africa and Asia. Front. Sustain. Food Syst..

[8] Tanumihardjo S.A., McCulley L., Roh R., Lopez-Ridaura S., Palacios-Rojas N., Gunaratna N.S. (2020). Maize agro-food systems to ensure food and nutrition security in reference to the Sustainable Development Goals. Glob. Food Secur..

[9] Bahar N.H., Lo M., Sanjaya M., Van Vianen J., Alexander P., Ickowitz A., Sunderland T. (2020). Meeting the food security challenge for nine billion people in 2050: What impact on forests?. Glob. Environ. Change.

[10] Singh A.K., Srivastava A.K., Johri P., Dwivedi M., Kaushal R.S., Trivedi M., Upadhyay T.K., Alabdallah N.M., Ahmad I., Saeed M. (2025). Odyssey of environmental and microbial interventions in maize crop improvement. Front. Plant Sci..

[11] Pimentel D., Whitecraft M., Scott Z.R., Zhao L., Satkiewicz P., Scott T.J., Phillips J., Szimak D., Singh G., Gonzalez D.O. (2010). Will limited land, water, and energy control human population numbers in the future?. Hum. Ecol..

[12] Munns R., Tester M. (2008). Mechanisms of salinity tolerance. Annu. Rev. Plant Biol..

[13] Kumar P., Choudhary M., Halder T., Prakash N.R., Singh V., Sheoran S., Longmei N., Rakshit S., Siddique K.H. (2022). Salinity stress tolerance and omics approaches in major cereal crops. Heredity.

[14] Ibrahim M.E.H., Zhu X., Zhou G., Ali A.A., Ahmad I., Farah G.A. (2018). Nitrogen fertilizer alleviates NaCl-induced physiological stress in wheat. Pak. J. Bot..

[15] Elsiddig A., Zhou G., Nimir N., Ali A. (2022). Effect of exogenous ascorbic acid on sorghum under salt stress. Chil. J. Agric. Res..

[16] Kancharla M., Madhu P., Prasuna P. (2025). Effect of saline irrigation on maize productivity and soil dynamics under drip fertigation. J. Soil Salin. Water Qual..

[17] Munns R. (2005). Genes and salt tolerance: Bringing them together. New Phytol..

[18] Xu Y., Yu Z., Zhang S., Wu C., Yang G., Yan K., Zheng C., Huang J. (2019). CYSTM3 negatively regulates salt stress tolerance in Arabidopsis. Plant Mol. Biol..

[19] Zhao J., Song H., Fan S., Zhou M., Zhao M. (2010). Growth response to ionic and osmotic NaCl stress in maize. J. Integr. Plant Biol..

[20] Ouhibi C., Attia H., Rebah F., Msilini N., Chebbi M., Aarrouf J., Urban L., Lachaal M. (2014). Salt stress mitigation by UV-C seed priming in lettuce. Plant Physiol. Biochem..

[21] De Angeli A., Zhang J., Meyer S., Martinoia E. (2013). AtALMT9 is a malate-activated vacuolar chloride channel required for stomatal opening. Nat. Commun..

[22] Colmenero-Flores J.M., Martínez G., Gamba G., Vázquez N., Iglesias D.J., Brumós J., Talón M. (2007). Cation–chloride cotransporters in plants. Plant J..

[23] Sandhu R., Irmak S. (2020). Performance of the Hybrid-Maize model under irrigation regimes. Agric. Water Manag..

[24] Wu H. (2018). Plant salt tolerance and Na^+^ sensing and transport. J. Integr. Plant Biol..

[25] Horie T., Hauser F., Schroeder J.I. (2009). HKT transporter-mediated salinity resistance mechanisms. Trends Plant Sci..

[26] Ismail A., Takeda S., Nick P. (2014). Life and death under salt stress. J. Exp. Bot..

[27] Galvan-Ampudia C.S., Julkowska M.M., Darwish E., Gandullo J., Korver R., Brunoud G., Haring M., Munnik T., Vernoux T., Testerink C. (2013). Halotropism enables roots to avoid saline environments. Curr. Biol..

[28] Franco-Navarro J.D., Brumós J., Rosales M.A., Cubero-Font P., Talón M., Colmenero-Flores J.M. (2016). Chloride regulates leaf cell size and water relations. J. Exp. Bot..

[29] Wu H., Li Z. (2019). Role of Cl^–^ transport in plant salt tolerance. Front. Plant Sci..

[30] Liang X., Li J., Yang Y., Jiang C., Guo Y. (2024). Designing salt-stress-resilient crops. J. Integr. Plant Biol..

[31] Parida A.K., Das A.B. (2005). Salt tolerance and salinity effects on plants: A review. Ecotoxicol. Environ. Saf..

[32] Rahneshan Z., Nasibi F., Moghadam A.A. (2018). Salinity effects on pistachio rootstocks. J. Plant Interact..

[33] Golldack D., Quigley F., Michalowski C.B., Kamasani U.R., Bohnert H.J. (2003). AKT1-type potassium channel regulation in rice. Plant Mol. Biol..

[34] Raza S.H., Athar H.R., Ashraf M., Hameed A. (2007). Glycine betaine-induced antioxidant modulation in wheat. Environ. Exp. Bot..

[35] Melgar J.C., Benlloch M., Fernández-Escobar R. (2006). Calcium increases sodium exclusion in olive plants. Sci. Hortic..

[36] Qu Y., Guan R., Yu L., Berkowitz O., David R., Whelan J., Ford M., Wege S., Qiu L., Gilliham M. (2022). Enhanced ROS detoxification in salt-stressed soybean roots. Physiol. Plant..

[37] Vennam R.R., Bheemanahalli R., Reddy K.R., Dhillon J., Zhang X., Adeli A. (2024). Early-season maize responses to salt stress. J. Agric. Food Res..

[38] Sudhir P., Murthy S.D.S. (2004). Effects of salt stress on photosynthesis. Photosynthetica.

[39] Sultan I., Khan I., Chattha M.U., Hassan M.U., Barbanti L., Calone R., Ali M., Majeed S., Ghani M.A., Batool M. (2021). Salicylic acid improves maize salinity tolerance. Ital. J. Agron..

[40] Zörb C., Noll A., Karl S., Leib K., Yan F., Schubert S. (2005). Molecular characterization of Na^+^/H^+^ antiporters (ZmNHX) of maize and their expression under salt stress. J. Plant Physiol..

[41] Shahid K., Liu Z., Shao L., Niu J., Chen S., Zhang X. (2025). Effects of manure application on soil physical properties and crop yield under long-term saline irrigation. Chin. J. Eco-Agric..

[42] Tian H., Liu H., Zhang D., Hu M., Zhang F., Ding S., Yang K. (2024). Screening of salt tolerance of maize lines using membership function value and GGE biplot analysis. PeerJ.

[43] Farooq M., Hussain M., Wakeel A., Siddique K.H.M. (2015). Salt stress in maize: Effects, resistance mechanisms, and management. Agron. Sustain. Dev..

[44] Hasanuzzaman M. (2020). Identification of yield predictors of wheat under salt stress using machine learning approaches. Int. J. Exp. Agric..

[45] Ding Z., Kheir A.M., Ali M.G., Ali O.A., Abdelaal A.I., Lin X.E., Zhou Z., Wang B., Liu B., He Z. (2020). Integrated effects of salinity, organic amendments, and irrigation on wheat productivity. Sci. Rep..

[46] Iqbal S., Hussain S., Qayyaum M.A., Ashraf M. (2020). Response of maize physiology to salinity stress and coping strategies. Plant Stress Physiology.

[47] Zhang Y., Li X., Wang J., Shi H., Chen N., Hu Q. (2023). Improved yield–salinity relationship considering salt and root distribution dynamics. Eur. J. Agron..

[48] Ezin V., Dasenka T., Ahanchede A., Handa A.K. (2025). Salt tolerance index and morpho-physiological characterization of tomato F_2_ segregating lines. Agric. Biosci..

[49] Fatima H., Khan A., Nadeem M., Khalofah A., Abbas T. (2025). Induction of salinity tolerance in maize by ascorbic and gibberellic acid application. Sci. Rep..

[50] Acosta-Motos J.R., Ortuño M.F., Bernal-Vicente A., Diaz-Vivancos P., Sanchez-Blanco M.J., Hernandez J.A. (2017). Plant responses to salt stress: Adaptive mechanisms. Agronomy.

[51] Hussein M.M., Balbaa L.K., Gaballah M.S. (2007). Salicylic acid and salinity effects on maize growth. Res. J. Agric. Biol. Sci..

[52] Carpici E.B., Celik N., Bayram G. (2010). Effects of salt stress on growth and mineral content of maize cultivars. Afr. J. Biotechnol..

[53] Shabala S., Munns R. (2017). Salinity stress: Physiological constraints and adaptive mechanisms. Plant Stress Physiology.

[54] Zhang D., Zhang Y., Sun L., Dai J., Dong H. (2023). Mitigating salinity stress and improving cotton productivity. Agronomy.

[55] Roy S.J., Negrão S., Tester M. (2014). Salt-resistant crop plants. Curr. Opin. Biotechnol..

[56] Masuda M.S., Azad M.A.K., Hasanuzzaman M., Arifuzzaman M. (2021). Evaluation of salt tolerance in maize at seedling stage. Plant Physiol. Rep..

[57] Abdel-Latef A.A.H., Sayed O.H. (2021). Salinity stress tolerance in maize through seed priming. Plants.

[58] Hasanuzzaman M. (2022). Salt stress tolerance in rice and wheat. Plant Defense Mechanisms.

[59] Rizk M.S., Mekawy A.M., Assaha D.V., Chuamnakthong S., Shalaby N.E., Ueda A. (2021). Regulation of Na^+^ and K^+^ transport reveals differential salt tolerance in maize hybrids. J. Plant Growth Regul..

[60] Rizk M.S., Assaha D.V., Mekawy A.M.M., Shalaby N.E., Ramadan E.A., El-Tahan A.M., Ibrahim O.M., Metwelly H.I., Okla M.K., Maridueña-Zavala M.G. (2024). Comparative analysis of salinity tolerance mechanisms in maize genotypes. BMC Plant Biol..

[61] Assaha D.V.M., Liu L., Mekawy A.M.M., Ueda A., Nagaoka T., Saneoka H. (2015). Salt stress effects on Na accumulation and antioxidant enzymes. Int. J. Agric. Biol..

[62] Mekawy A.M.M., Assaha D.V., Yahagi H., Tada Y., Ueda A., Saneoka H. (2015). Growth and gene expression of rice under salt stress. Plant Physiol. Biochem..

[63] Mekawy A.M.M., Assaha D.V., Ueda A. (2020). Differential salt sensitivity of flax cultivars. J. Plant Growth Regul..

[64] Iftikhar N., Perveen S., Ali B., Saleem M.H., Al-Sadoon M.K. (2024). Physiological and biochemical responses of maize to salinity stress. Turk. J. Agric. For..

[65] Rizk M.S., Assaha D.V., Mekawy A.M.M., Shalaby N.E., Ramadan E.A., El-Tahan A.M., Ibrahim O.M., Metwelly H.I., Okla M.K., Maridueña-Zavala M.G. (2024). Comparative traits in maize genotypes under salinity and Pb stress. Sci. Rep..

[66] Ahmad S., Cui W., Kamran M., Ahmad I., Meng X., Wu X., Su W., Javed T., El-Serehy H.A., Jia Z. (2021). Melatonin-induced salt stress tolerance in maize seedlings. J. Plant Growth Regul..

[67] Hiscox J.D., Israelstam G.F. (1979). Chlorophyll extraction without maceration. Can. J. Bot..

[68] Arnon D.I., Whatley F.R. (1949). Is chloride a coenzyme of photosynthesis?. Science.

[69] Lichtenthaler H.K., Welburn A.R. (1983). Determination of carotenoids and chlorophylls. Biochem. Soc. Trans..

[70] Munns R., Wallace P.A., Teakle N.L., Colmer T.D. (2010). Measuring soluble ion concentrations. Plant Stress Tolerance.

[71] Fox J., Friendly G.G., Graves S., Heiberger R., Monette G., Nilsson H., Ripley B., Weisberg S. (2007). The car package. R Found. Stat. Comput..

[72] Lê S., Josse J., Husson F. (2008). FactoMineR: An R package for multivariate analysis. J. Stat. Softw..

[73] Wickham H. (2016). Getting Started with ggplot2. ggplot2: Elegant Graphics for Data Analysis.

[74] Pour-Aboughadareh A., Yousefian M., Moradkhani H., Moghaddam Vahed M., Poczai P., Siddique K.H. (2019). iPASTIC: An online toolkit to estimate plant abiotic stress indices. Appl. Plant Sci..

[75] Fischer R.A., Maurer R. (1978). Drought resistance in spring wheat cultivars. I. Grain yield responses. Aust. J. Agric. Res..

[76] Rosielle A.A., Hamblin J. (1981). Theoretical aspects of selection for yield in stress and non-stress environments. Crop Sci..

[77] Fernandez G.C.J., Kuo C.G. (1992). Effective selection criteria for assessing plant stress tolerance. Proceedings of the International Symposium on Adaptation of Vegetables and Other Food Crops in Temperature and Water Stress.

[78] Bouslama M., Schapaugh W.T. (1984). Stress tolerance in soybeans. Part 1: Evaluation of three screening techniques for heat and drought tolerance. Crop Sci..

